# Application of Skeleton Data and Long Short-Term Memory in Action Recognition of Children with Autism Spectrum Disorder

**DOI:** 10.3390/s21020411

**Published:** 2021-01-08

**Authors:** Yunkai Zhang, Yinghong Tian, Pingyi Wu, Dongfan Chen

**Affiliations:** 1 School of Communication and Electronic Engineering, East China Normal University, Shanghai 200241, China; 51191213074@stu.ecnu.edu.cn (Y.Z.); yhtian@cee.ecnu.edu.cn (Y.T.); 2 Experimental Teaching Center for Teacher Education, East China Normal University, Shanghai 200241, China; 3 Department of Rehabilitation Sciences, East China Normal University, Shanghai 200062, China; dfchen@spe.ecnu.edu.cn

**Keywords:** ASD children, action recognition, LSTM, skeleton data

## Abstract

The recognition of stereotyped action is one of the core diagnostic criteria of Autism Spectrum Disorder (ASD). However, it mainly relies on parent interviews and clinical observations, which lead to a long diagnosis cycle and prevents the ASD children from timely treatment. To speed up the recognition process of stereotyped actions, a method based on skeleton data and Long Short-Term Memory (LSTM) is proposed in this paper. In the first stage of our method, the OpenPose algorithm is used to obtain the initial skeleton data from the video of ASD children. Furthermore, four denoising methods are proposed to eliminate the noise of the initial skeleton data. In the second stage, we track multiple ASD children in the same scene by matching distance between current skeletons and previous skeletons. In the last stage, the neural network based on LSTM is proposed to classify the ASD children’s actions. The performed experiments show that our proposed method is effective for ASD children’s action recognition. Compared to the previous traditional schemes, our scheme has higher accuracy and is almost non-invasive for ASD children.

## 1. Introduction

Autism, or Autism Spectrum Disorder (ASD), is a neurodevelopmental disorder, characterized by persistent deficits in social communication and interaction as well as restricted and repetitive behaviors [1]. The cause of ASD is extremely complex, involving genetic and environmental factors [2,3]. In recent years, the number of children diagnosed with ASD has been increasing [4,5]. It is estimated that one in 160 children have autism [6]. This puts great pressure on society.

Studies have shown that early diagnosis and intervention are the most effective clinical treatment methods for ASD [7,8]. At present, the diagnosis of ASD is complicated and mainly relies on diagnostic tools, including parent interviews and clinical observations. These measures have an extend period, leading to delays in ASD intervention and treatment, causing ASD children to miss the window period. Moreover, parents of ASD children will be under tremendous pressure and lose confidence in the relevant professional medical staff after a long diagnosis delay [9]. Howlin et al. [10] surveyed over 1200 parents of ASD children and found that about half of the families were “not too” or “not at all” satisfied with the ASD diagnosis process. Studies have confirmed that dyskinesias are common in children with ASD [11,12,13]. They often have uncontrolled repetitive actions, such as clapping hands, shaking the body, and repeatedly fiddling with toys and objects. In the United States (DSM–V) [1] and Europe (ICD–10) [14], stereotyped action is the core diagnostic criteria in the professional clinical practice of ASD. Therefore, accurate and rapid recognition of ASD children’s action is an important idea to accelerate diagnosis.

For the action recognition of ASD children, direct observation is the most widely used method [15,16,17], i.e., rehabilitation experts directly observe the behavior of ASD children, then record and analyze their stereotyped actions [18]. However, ASD children move swiftly, rehabilitation experts cannot accurately observe and record all stereotyped actions. It is also difficult to determine the start and end time.

In addition to direct observation, wearable sensor-based methods provide a promising solution for action recognition in ASD children. Before the Deep Neural Network (DNN) is fully developed [19,20,21], manual extraction of features from sensor data for action classification is the most common. Gonçalves et al. [22] used the acceleration sensor worn on the right arm of ASD patients to collect action’s data and then analyzed the statistical features such as mean, variance, peak number, and root mean square to detect the stereotyped actions. Crippa et al. [23] designed a simple ball-grabbing task. They used an optoelectronic system to obtain the kinematics data of ASD children when they completed the actions such as reach, grasp, and drop, then extracted 17 kinematics indicators as features. Finally, the SVM classifier was used to classify different actions. The above works were designed to quickly and accurately obtain the action information of ASD children from wearable sensor data. The sensor’s advantages are simplicity, stability, and high sensitivity, but the disadvantages are also obvious. On the one hand, the sensor cannot analyze the complex actions of the human body. On the other hand, it is too invasive for ASD children. Wearing sensors will distract them, which will change their behavior and affect the accuracy of their actions [24,25]. Moreover, manual selection and extraction of features rely on researchers’ professional knowledge, and the omission of essential features related to the task will cause the system to fail. Compared with the wearable sensor-based methods, video-based automatic analysis methods are almost non-invasive. They have been widely used with the development of computer vision technology, such as football videos [26,27], basketball videos [28,29], tennis videos [30,31], and taekwondo videos [32,33]. However, due to the uncertainties and noisy backgrounds of ASD children’s action, there are few attempts to analyze ASD children’s videos.

In recent years, with the application of deep learning in the biomedical field [34,35,36,37], especially the development of Deep Neural Network and hardware computing capabilities, a suitable method is to use neural networks to extract action information from multi-dimensional data and integrate them into the action recognition of ASD children. Rad et al. [38] used a three-layer CNN network to automatically extract features from ASD patients’ data collected by acceleration sensors and ordinary inertial measurement units (IMU), then they used an SVM classifier for classification. The results showed that neural networks are superior to traditional manual feature extraction methods. Cook et al. [39] used the OpenPose algorithm to extract the skeleton data of ASD children’s upper limbs from the RGB image, then they calculated each key point’s speed to extract the features manually. Their method could recognize clapping, swinging back and forth, and repeatedly playing with the toy. They achieved 71% accuracy using the Decision Tree as a classifier. The results of various studies show that the action recognition of ASD children is still a challenging problem.

Considering this, to accelerate the diagnosis process, this paper proposes an effective ASD children action recognition method. Our method uses the OpenPose [40] algorithm to extract the skeleton data of ASD children from the video and then recognizes the action through the Long Short-Term Memory networks (LSTMs). The main contributions of this paper include:

1. We propose four measures to eliminate the initial skeleton data’s noise, which improve recognition accuracy and calculation efficiency.
2. The multi-person tracking method based on skeleton data is proposed to track multiple ASD children in the video. Unlike the latest multiple objects tracking technologies, our method does not need additional GPUs and is suitable for multi-person tracking in a fixed scene.
3. An action recognition model based on LSTMs is proposed in this paper, and the end-to-end deep learning-based framework eliminates the need to extract features manually. The experimental results show that the Precision, Recall, and F1-score are improved, and the proposed model outperforms other manual feature extraction-based methods on our ASD children dataset.
4. We evaluate the impact of the input data time steps and the number of hidden states on the LSTM network’s accuracy, which is crucial for the recognition of ASD children’s actions.

The rest of this paper is organized as follows: Section 2 introduces the proposed method, including three parts: the generation of de-noised skeleton data for ASD children, multi-person tracking based on skeleton data, and ASD children’s action recognition based on LSTM network. Section 3 describes the experiments and results, mainly including the introduction of the experimental dataset, evaluation of important parameters, system performance evaluation, and comparison with other methods. Finally, conclusions are drawn in Section 4.

## 2. Proposed Method

**Figure 1:**
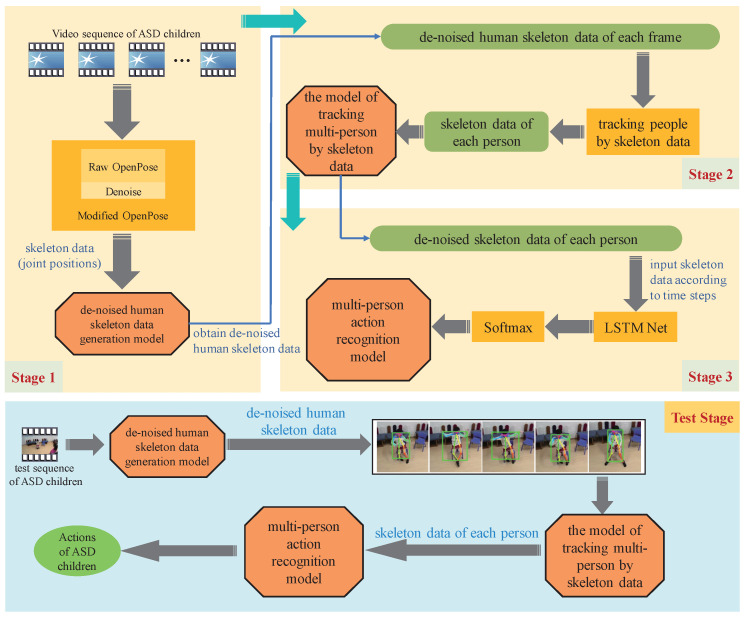
Flow chart of proposed ASD children action recognition method.

The flow chart of our ASD children action recognition method is shown in Figure 1. It consists of three stages. In stage 1, the OpenPose algorithm obtains the skeleton data of ASD children in the video sequence. However, the original skeleton data will be lost due to occlusion or overlap. Therefore, we denoise the skeleton data by discarding frames with missing important information and deleting the head data that has little effect on this task (detailed in Section 2.1). In stage 2, to track multi-person in real time, the skeleton data in different frames obtained by stage 1 are matched to obtain each person’s continuous skeleton data (detailed in Section 2.2). Finally, each ASD children’s skeleton data obtained by stage 2 as the input of the LSTM model to recognize actions. With all stages finish, we finally achieve three models: de-noised human skeleton data generation model, multi-person tracking model based on skeleton data, and multi-person action recognition model.

**Figure 2:**
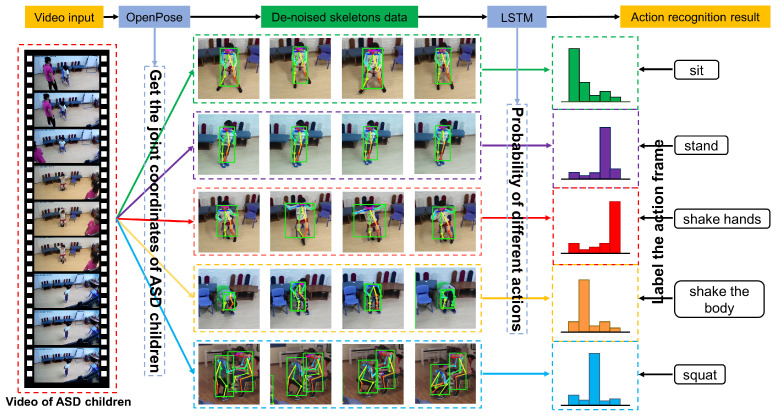
Our method first extracts the de-noised skeleton data (body key points coordinates) of ASD children in the video sequence and then analyzes the skeleton data through the LSTM network. Finally, the actions of ASD children are divided into five categories.

Based on these three models, we build a system for ASD children’s action recognition. To make our flow chart clearer, Figure 2 shows the visual flow of the system recognizing five different actions.

### 2.1. Generation of De-Noised Human Skeleton Data

In this paper, OpenPose [40] is used to obtain original human skeleton data. However, there are two problems with the original skeleton data: On the one hand, ASD children are sometimes occluded, which leads to a lack of skeleton data. On the other hand, ASD children’s actions are highly random, making the acquired skeleton data not obvious, and it is difficult to recognize the actions accurately. For this, we have taken four measures to eliminate noise: First, we scale the coordinates of the key points outputted by OpenPose to the same unit. Second, we remove the five joints on the head. Third, we discard frames without skeleton data or missing important joints. Finally, we use the relative joint positions in adjacent frames to fill in the unrecognized joint positions.

**Figure 3:**
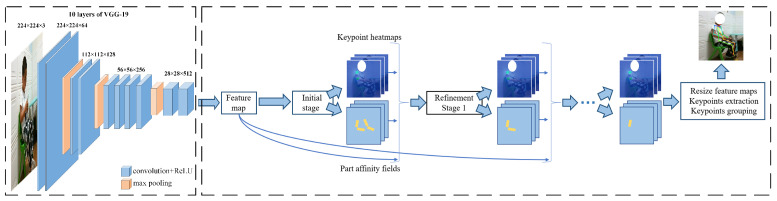
The pipeline of OpenPose.

**Figure 4:**
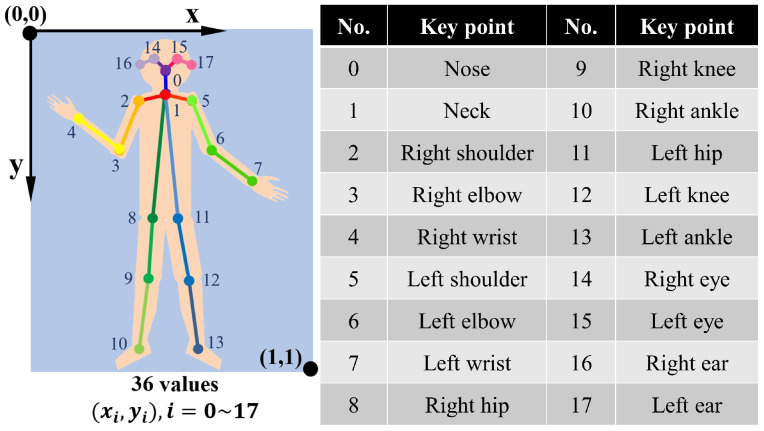
Key points detected using OpenPose.

Figure 3 illustrates the overall pipeline of OpenPose. The system takes, as input, a color image of size w×h and produces, as output, the 2D location of anatomical key points for each person in the image. It uses the first 10 layers of VGG-19 [41] as a feature extractor, generating a set of feature maps F that is input to a feedforward network. First, the feedforward network simultaneously predicts a set of 2D confidence maps S of body part locations and a set of 2D vector fields L of part affinities, which encode the degree of association between parts. The set $S=(S_{1},\;\;S_{2},\;\ldots ,\;\;S_{J})$ has *J* confidence maps, one per part, where $S_{j}\in R^{w},j\in \left\{1\ldots J\right\}$. The set $L=(L_{1},\;\;L_{2},\;\ldots ,\;\;L_{C})$ has *C* vector fields, one per limb, where $L_{c}\in R^{w\times 2},c\in \left\{1\ldots C\right\}$, each image location in $L_{c}$ encodes a 2D vector. Finally, the confidence maps and the affinity fields are parsed by greedy inference to output the 2D key points for all people in the image. The output includes ears, eyes, nose, neck, shoulders, elbows, wrists, knees, hips, and ankles, as shown in Figure 4.

**Figure 5:**
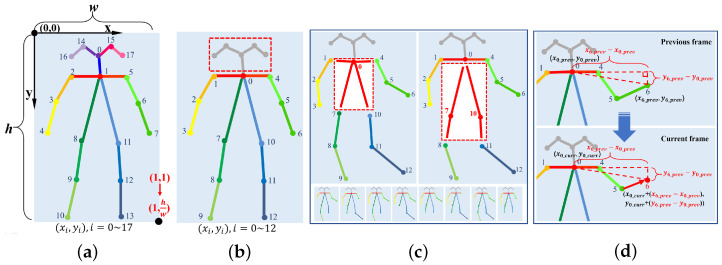
Proposed four de-noised measures. (**a**) Scale the co-ordinate. (**b**) Remove all joints on the head. (**c**) Discard frames without Neck or Hip. (**d**) Fill in the missing joints.

The original joint positions outputted by OpenPose has a different unit for the X co-ordinate and Y co-ordinate. As shown in Figure 5a, we scaled them to the same unit to deal with the images with different height/width ratios. Moreover, the head’s position helps little for the action classification. What matters is the configuration of the body and limbs. Thus, we manually removed the five joints (Nose, Right eye, Left eye, Right ear, Left ear) on the head, and the Neck becomes the 0th joint, as shown in Figure 5b. If in a frame there is no human skeleton detected by OpenPose or the detected skeleton has no Neck or Thigh (top of Figure 5c), then this frame is considered invalid and will be discarded. On the contrary, frames missing other joints will be retained (bottom of Figure 5c).

In some cases, OpenPose might fail to detect a complete human skeleton from the image, causing some blank in the joint positions. These joints must be filled with some values to maintain a fixed position for the following automatic feature extraction procedure. Two bad solutions are:

1. Discard this frame. However, in this way, the algorithm will barely recognize the action when the person is not facing the camera.
2. Fill in the joint positions with some value outside a reasonable range. In theory, when the algorithm is strong enough, this method could work. However, this requires a lot of experimentation and more calculations.

In this paper, we will set the missing joints as their relative positions to the Neck in the previous frame. Suppose a joint $\left(x_{i},y_{i}\right)$ is missing in the current frame, the position of it can be expressed as:

(1) $$x_{i\_curr}=x_{Neck\_curr}+\left(x_{i\_prev}-x_{Neck\_prev}\right),$$

(2) $$y_{i\_curr}=y_{Neck\_curr}+\left(y_{i\_prev}-y_{Neck\_prev}\right),$$

Figure 5d shows an example of using our method to fill in the missing joint.

The four measures to eliminate noise are described above. It is noteworthy that the skeleton data we currently obtain is for everyone in a frame. Therefore, a multi-person tracking technique should be used to obtain different persons’ id in a frame and match their skeleton data.

### 2.2. Tracking Multi-Person by Skeleton Data

In an ASD rehabilitation scenario, there are usually multiple children. So, tracking them is needed. It should be mentioned that for multiple object tracking (MOT), a usual consideration is that some commonly used algorithms such as SORT [42] and Deep SORT [43]. Through the calculation of the Convolutional Neural Network (CNN), they can achieve good accuracy. However, these methods have a high computational burden, which is hardly put into a real-time system. In this paper, we design an algorithm to track multiple people through human skeleton data in video sequences.

First, after the skeleton data is de-noised in Section 2.1, the multi-person skeleton data $S_{n}$ of each frame is obtained. Any two consecutive frames can be expressed as previous skeleton data $S_{prev}$ and current skeleton data $S_{curr}$. The skeleton data of each person in each frame is expressed as:

(3) $$S_{prev}\left[i\right]=\left\{\left(x_{i\_0},y_{i\_0}\right),\left(x_{i\_1},y_{i\_1}\right),...,\left(x_{i\_12},y_{i\_12}\right)\right\},$$

(4) $$S_{curr}\left[j\right]=\left\{\left(x_{j\_0},y_{j\_0}\right),\left(x_{j\_1},y_{j\_1}\right),...,\left(x_{j\_12},y_{j\_12}\right)\right\},$$

where i,j represents different people in previous and current frame, respectively. Then, all skeletons are sorted based on the distance between the neck $\left(x_{0},y_{0}\right)$ and the image center $\left(x_{center},y_{center}\right)$, from small to large. This step provides convenience for matching skeletons between current and previous. The calculation formula is as follows:

(5) $$D=\sqrt{{\left(x_{0}-x_{center}\right)}^{2}+{\left(y_{0}-y_{center}\right)}^{2}}.$$

It should be noted that if $S_{prev}$ is the first frame containing skeleton data, then the human id of everyone will be initialized according to the distance from the center of the frame.

Finally, matching the distance between current skeletons and previous skeletons. If $S_{curr}\left[j\right]$ and $S_{prev}\left[i\right]$ are matched, for $S_{prev}\left[i\right]$, $S_{curr}\left[j\right]$ is the nearest skeleton in $S_{curr}$ and for $S_{curr}\left[j\right]$, $S_{prev}\left[i\right]$ is the most nearest skeleton in $S_{prev}$ as well. Moreover, the distance between the two matched people’s joints should be smaller than our pre-set threshold. For unmatched skeletons in $S_{curr}$, they are considered to be new people appeared in the video.

Through the above steps, we can track the input skeletons by matching them with previous skeletons and then obtain their corresponding human id.

### 2.3. Action Recognition Using LSTM Net

The skeleton data of different actions are time series. In this paper, the Long Short-Term Memory (LSTM) network automatically extracts features from skeleton data. The LSTM [44] is a special type of Recurrent Neural Network (RNN), which adds prior knowledge in the hidden layer: input gate, forget gate, and output gate. These gates process the inter-layer information at different moments and the input information at a certain moment more transparently, which can effectively pass the past information to the current calculation and can overcome the defect that the RNN structure cannot pass far apart information [45].

**Figure 6:**
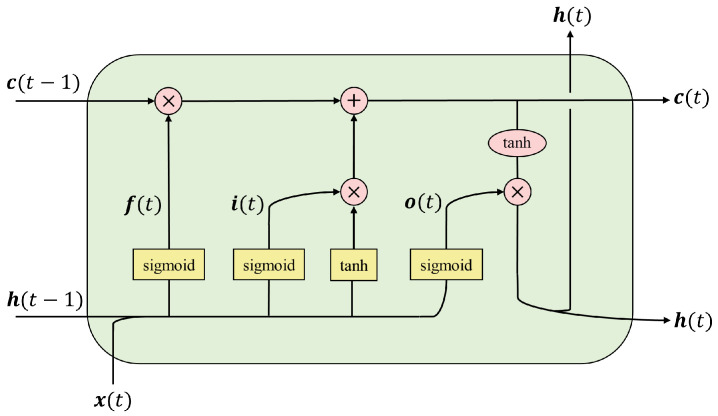
Structure diagram of the LSTM unit.

The structure of the LSTM unit is shown in Figure 6, the function expressions of input gate, forget gate, and output gate can be obtained as follows:

(6) $$i\left(t\right)=sigmoid(W_{xi}x\left(t\right)+W_{hi}h\left(t-1\right)+b_{i}),$$

(7) $$f\left(t\right)=sigmoid(W_{xf}x\left(t\right)+W_{hf}h\left(t-1\right)+b_{f}),$$

(8) $$o\left(t\right)=sigmoid(W_{xo}x\left(t\right)+W_{ho}h\left(t-1\right)+b_{o}),$$

where $W_{x}$ is the input weight matrix, $W_{h}$ is the hidden layer state weight matrix at time t−1, b is the bias term. The self-connected unit state c(t) and hidden layer state h(t) at time *t* are expressed as:

(9) $$\begin{array}{cc} c(t)= & f\left(t\right)\cdot c\left(t-1\right)+i\left(t\right)\cdot \tanh\left(W_{xc}x\left(t\right)+W_{hc}h\left(t-1\right)+b_{c}\right), \end{array}$$

(10) h(t)=o(t)·tanh(c(t)).

Analyzing (6)–(10), it can be found that by adjusting the weight matrix W of each gate, the input gate i(t) can control the amount of information flowing into the self-connected unit state c(t), and the forget gate f(t) can control the amount of information c(t−1) contained in the self-connected unit state c(t) at the current moment, i.e., how much information is forgotten. The output gate o(t) controls the information of self-connected unit state c(t) that can flow into the current hidden layer state h(t). Among them, the role of the self-connected unit state c(t) is to complete the accumulation of historical information, and its accumulation method is:

(11) $$Set:info=\tanh(W_{xc}x\left(t\right)+W_{hc}h\left(t-1\right)+b_{c}),$$

where info is the source of information to be accumulated, substituting (11) into (9) to obtain:

(12) c(t)=f(t)·c(t−1)+i(t)·info.

From (12), it can be known that when the self-connected unit state c(t) accumulates historical information, it relies on the forget gate f(t) to limit the information transmitted at the previous moment c(t−1), and at the same time, the input gate i(t) constrain the newly entered information. According to (10), the current hidden layer state h(t) is constrained by the output gate. Since it is updated linearly, the tanh function with nonlinear is added.

The information source of the entire LSTM unit is the current input x(t), the hidden layer state h(t−1) at the previous moment, and the linear self-connected unit state c(t−1) at the previous moment. Since c(t−1) is calculated according to (9), the control basis of the three gate units actually comes from the current input x(t) and the hidden layer state h(t−1) at the previous moment.

Compared with the traditional methods [22,23,46], using a Recurrent Neural Network (RNN) with Long short-term memory (LSTM) units requires almost no feature engineering, and the data can be directly fed into the neural network to model the problem correctly.

**Figure 7:**
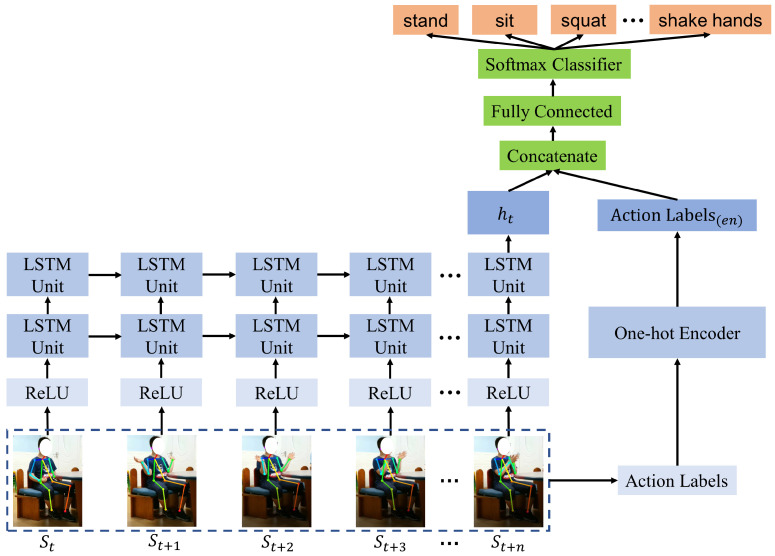
Schematic diagram of the ASD children action recognition network.

Figure 7 shows the action recognition network used in our method. The input skeleton data has a shape of n × 13 × 2, which denotes the n sequential frames with 13 key points having X and Y coordinates each. First is a fully connected layer activated by ReLU, and then two stacked LSTM layers are applied to the skeleton data of each frame. A many-to-one architecture of LSTM is used in this paper. LSTM leverages the sequential nature of the input sequences to identify temporal changes in skeleton data. The LSTM layers’ output is passed to a fully connected layer with SoftMax activation and five outputs. Each of these five outputs provides the probability of the corresponding action in terms of cross-entropy. Thresholding is applied to this output to detect when the ASD children are performing specific actions.

## 3. Experiments and Results

In this section, we first introduce the ASD children dataset used in this paper. Next, two important parameters are evaluated. Then, we verify the effectiveness of the proposed skeleton data de-noised methods. Finally, we compare the proposed approach with several methods of manually extracting features.

### 3.1. Experiment Dataset

**Figure 8:**
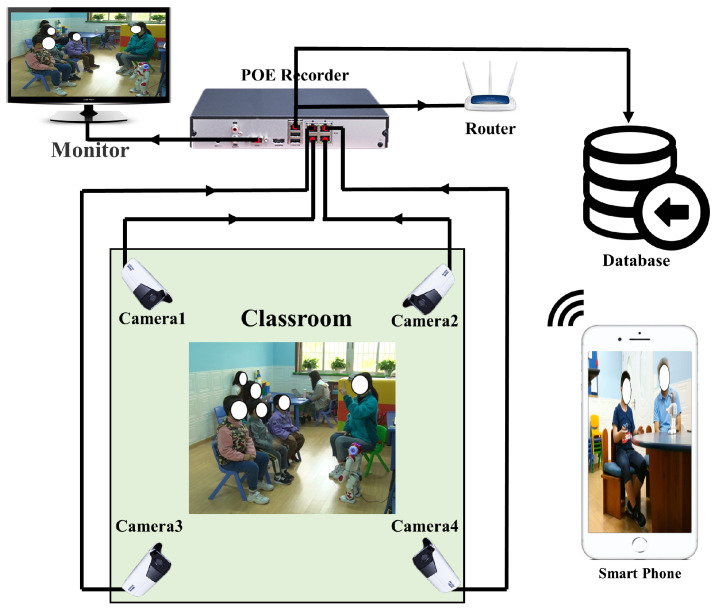
Data collection system.

There is currently no public dataset that can be used for the action recognition of ASD children. The dataset used in this paper was collected in our cooperative ASD rehabilitation institution. We arranged four 5 million pixels HIKVISION remote cameras in the classroom to collect videos of ASD children from four different angles, then transferred the images to the database through the POE recorder. We can view the real-time situation in the classroom through a smart phone, and adjust the shooting angle of the camera remotely to ensure that the image with the best angle can be collected without disturbing the ASD children. The data collection system is shown in Figure 8.

Our purpose was to collect ASD children’s videos in various real environments, to mark some stereotyped actions. The children with ASD selected by us are about 5 to 10 years old. It is worth noting that not all children are severely autistic, some children have milder symptoms, and their actions are similar to normal children. We mainly focused on their repetitive actions. These actions are usually very fast, such as shaking hands, shaking their bodies, and standing up suddenly, they are easily ignored by ASD children’s busy parents.

In this paper, the dataset we made contains video clips of 5 actions: sit, stand, squat, shake the body, and shake hands. Each clip is a sequence of images about 1–3 s (25 frames per second), a total of 1062 sequences. Each sequence contains the complete process of a single action. Table 1 below describes an overview of the dataset, including the name of each action, the number of frames, and the number of sequences.

After obtaining the dataset, we normalized the image size to 656 × 368, then sent them to the de-noised human skeleton data generation model proposed in Section 2.1. It should be noted that when extracting skeleton data for training the LSTM network, there may be multiple ASD children in some frames. For this, we only keep the skeleton data of the ASD child closest to the image center.

### 3.2. Evaluation of Parameters

Model network structure and the input data structure have a fundamental impact on the effect of action recognition. In this section, two important parameters that affect the accuracy of the LSTM network were evaluated. One is the time steps of the input sequence, which indicates how many frames constitute an action sequence. It is related to the structure of the input data. The other is the number of hidden states of the LSTM network, which is related to the network structure.

**Figure 9:**
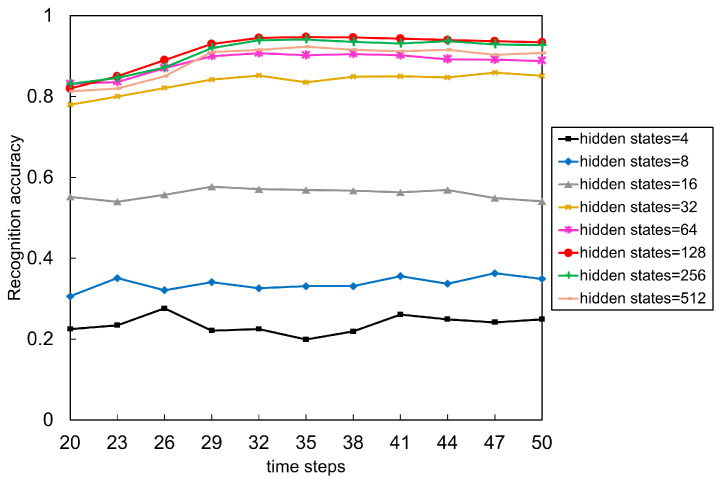
The recognition accuracy of the LSTM model under different time steps and hidden states.

The time steps of the input sequence is one of the important parameters in making a training dataset. On the one hand, action sequences with small time steps cannot guarantee that it covers all the process of an action. On the other hand, action sequence with too large time steps will not only increase the pressure of network computing, but also introduce some interference features affecting action recognition of ASD children. Thus, to verify the effect of input data structure on network performance, datasets with time steps from 20–50 were trained under other conditions unchanged. As shown in Figure 9.

The number of hidden states, one of the basic parameters of the LSTM network structure, represents the number of nodes used to remember and store the past state, which determines how much information is remembered and how much is forgotten. From the construction principle of the neural network, the more hidden states, the more data features and the higher adaptability of the network. However, too many hidden states will overfit the neural network. Therefore, the extracted dataset was used to test the impact of the number of hidden states on recognition accuracy. As shown in Figure 9.

The extracted dataset from the ASD children dataset we made in Section 3.1, including 151 sequences of sit, 149 sequences of stand, 133 sequences of squat, 150 sequences of shake the body, and 141 sequences of shake hands. 70% of the complete action sequences were used to train the model, and the remaining 30% were used to test the recognition model. It is worth noting that in the original dataset proposed in Section 3.1, the length of a single action is 1–3 s (25–75 frames). Therefore, to evaluate the effect of the sequence’s time step, the original action sequence should be scaled to 20–50 frames. The methods we used as following:

- For the sequences that are less than 20 frames, we fill them with all-zero skeleton data at the end of the sequence.
- For the sequences that are more than 50 frames, we randomly select frames without repeating and make them form a shortened frame sequence in the original order.

According to Figure 9, we first analyze the impact of time steps on recognition accuracy. When the number of hidden states is 4 to 16, the network is too simple. At this time, the time steps have almost no effect on the accuracy of the model. When the number of hidden states between 32 and 512, increasing the time steps from 20 to 32 improves the model’s accuracy, and the accuracy almost unchanged after more than 32 frames. The decrease in accuracy at a time steps of 50 means that the time span is so long that the model learns the interference features. The above results indicate that for our dataset, the time steps of 32 frames (about 1.3 s) can summarize the action features of ASD children.

Then analyze the impact of hidden states, when the number of hidden states from 4 to 16, the network structure is so simple that the model cannot learn enough features from the data. At this time, the accuracy is low. When the number of hidden states from 32 to 512, the accuracy of the model improves significantly, which indicates that the network has learned more features to classify actions. When the number of hidden states is 128, the model reaches the best accuracy. It is worth noting that when the number of hidden states changes from 256 to 512, the accuracy decreases, mainly due to the over-fitting phenomenon caused by the complex network structure, which significantly reduces the generalization ability of the model.

Considering these factors comprehensively, we got the following best parameters: the time steps is 32, the number of hidden states is 128, and the learning rate is 0.001.

### 3.3. Performance Evaluation

To verify the impact of the skeleton data de-noised methods proposed in Section 2.1, we used the ASD children dataset to experiment with different time steps and the number of hidden states.

**Figure 10:**
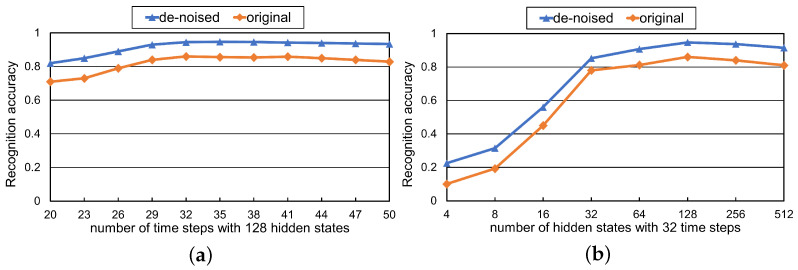
Action recognition accuracy of the model under original and de-noised skeleton data. (**a**) Varying the number of time steps. (**b**) Varying the number of hidden state.

In the experiment, we selected 749 action sequences from the ASD children dataset made in Section 3.1 (144 for sit, 151 for stand, 129 for squat, 171 for shake the body, and 154 for shake hands). 70% of the complete sequences are used for training, and the remaining 30% are used for testing. After processing, we got two groups of skeleton data. One group was obtained after denoising using proposed methods, and the other group was the original skeleton data extracted by OpenPose. The results are shown in Figure 10. After denoising, the recognition accuracy under different time steps and hidden states is higher than that of unprocessed original data, about 10%. It shows that our skeleton data de-noised methods can make the model learn more useful features and reduce the influence of interference features. It is worth noting that same as the best parameters in Section 3.2, the model achieves a balance between recognition accuracy and calculation pressure under the time steps of 32 and the hidden state of 128, which shows that the best parameters obtained are indeed suitable for our experiments.

**Figure 11:**
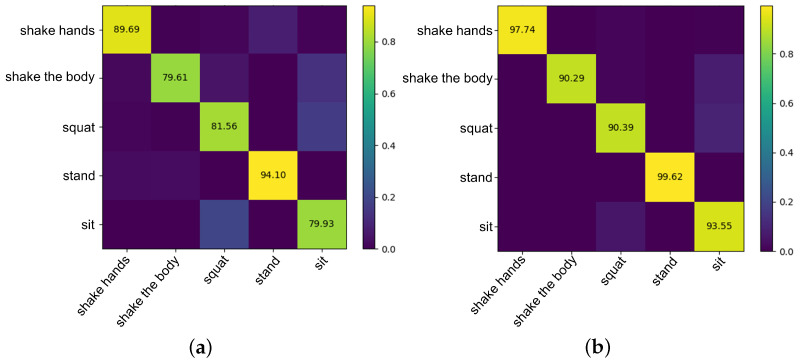
Confusion matrices obtained from original and de-noised skeleton data using the best parameters. (**a**) The matrices with original skeleton data. (**b**) The matrices with de-noised skeleton data.

Two kinds of confusion matrices, obtained from original and de-noised skeleton data using the best parameters, are presented in Figure 11. The row of a confusion matrix represents true classes of samples while the column represents the predicted classes. The diagonal entries represent the true classification rate of each class, the shallower the color is, the higher the true classification rate is. Figure 11a is the confusion matrix obtained from the original skeleton data extracted by OpenPose, while Figure 11b uses the de-noised skeleton data. Obviously, skeleton data denoising processing improves action recognition accuracy, especially shake the body, squat, and sit. The features of these three actions are more complex. However, the denoising processing enables the network to learn more useful features and reduces invalid features’ interference. It is worth noting that for the two experiments, shake the body was confused with sit. A very important reason is that in our dataset, ASD children shaking the body are done in a sitting position, so the features of these two actions are similar. In this case, our denoising methods achieves a recognition rate of 90.29%. Similarly, squat and sit have been misrecognized due to decreased body height, but our denoising method finally gets a recognition rate of 90.39% and 93.55%, respectively.

**Figure 12:**
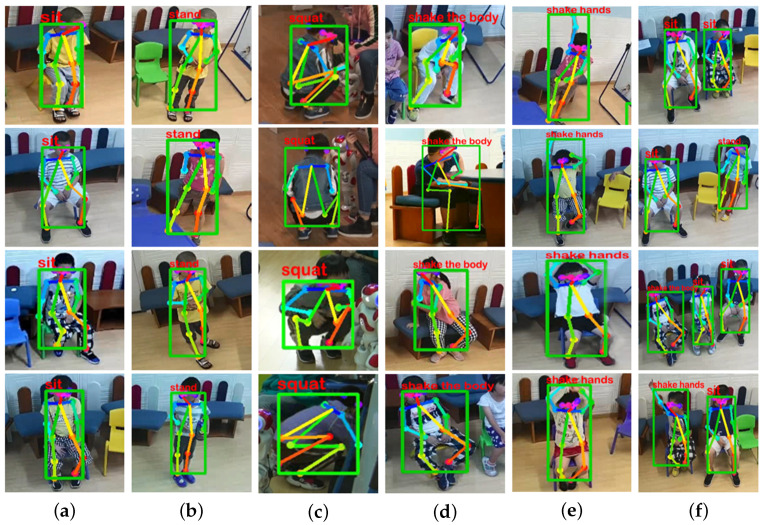
Sample images in real-time tests. (**a**) Sit. (**b**) Stand. (**c**) Squat. (**d**) Shake the body. (**e**) Shake hands. (**f**) Multi-person.

Figure 12 shows some screenshots from real-time tests. Through the multi-person tracking method based on skeleton data proposed in Section 2.2, our system supports real-time action recognition of multiple ASD children. The threshold we set is 0.9. When the probability of action is greater than 0.9, the system will determine that the ASD children are performing this action and display the label of the action on the character selection box.

### 3.4. Comparison and Analysis

To further verify the accuracy of the proposed method, manual feature extraction-based methods were introduced to recognize ASD children’s actions. In the experiments, we selected 844 action sequences from the ASD children dataset made in Section 3.1 (160 for sit, 155 for stand, 151 for squat, 190 for shake the body, 188 for shake hands). 70% of the complete sequences were used for training and the remaining 30% were used for testing.

Manual feature extraction is very popular in motion analysis. In the field of ASD children’s action recognition, the most common is to extract features from sensor data. Gonçalves et al. [22] and Albinali et al. [47,48,49] both used acceleration sensors to collect action data, then captured time and frequency domain features from them, finally recognized the actions of ASD children. With the development of human pose estimation technology based on RGB images (such as OpenPose and AlphaPose [50]), extracting features from skeleton data can also achieve good performance. Like our work, [39] used OpenPose to extract the skeleton data of ASD children and then manually extract features from it for action recognition.

To compare the recognition effects of the proposed LSTM-based method and manual feature extraction methods more comprehensively, we re-set two feature extraction methods. Method 1 was based on [39], and the extracted features included the maximum magnitude, mean magnitude, and standard deviation of the autocorrelogram maxima. These features were calculated from each key point’s three velocity measures (velocity change in the X-axis, Y-axis, and overall position). It should be noted that [39] only used six key points of the upper body to extract features: Right shoulder, Right elbow, Right wrist, Left shoulder, Left elbow, and Left wrist. It means that their method was only sensitive to the upper body actions of ASD children. However, our dataset includes some lower body actions, so based on the original features, we added all normalized joints except the head positions as additional features to ensure the effectiveness of action recognition.

Method 2 included three features: normalized joint positions *X*, moving velocity of the body $V_{body}$, and joint velocities $V_{joints}$. Compared with method 1, these features contained information on all key points and can better summarize the actions of ASD children. A summary of the computed features is shown in Table 2. In addition, method 1 used the Decision Tree mentioned in [39] as classifier, and method 2 used DNN (3 layers, 100 × 100 × 100), Linear SVM, and Random Forests (depth 40, trees 100) as classifiers. The implementation of these classifiers was from the Python library “sklearn”.

**Figure 13:**
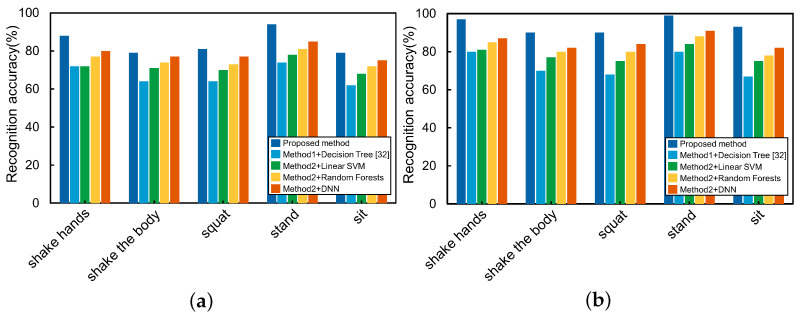
Action recognition accuracy of five different methods.(**a**) Using original skeleton data. (**b**) Using de-noised skeleton data.

The experiments were carried out under the original and de-noised skeleton data, respectively. Figure 13 shows the recognition accuracy of different methods. Simultaneously, to further verify the performance of the proposed method, Table 3 reports the Precision, Recall, and F1-score of each action computed over all the methods, which are defined as follows:

(13) $$Precision:\;P=\frac{TP}{TP+FP},$$

(14) $$Recall:\;R=\frac{TP}{TP+FN},$$

(15) $$F1-score=\frac{2\times P\times R\;}{P+R\;},$$

where True Positive (TP) represents the number of actions correctly recognized, False Positives (FP) represents the number of actions falsely recognized, and False Negatives (FN) represents the number of missed actions.

By analyzing the performance indicators in Figure 13 and Table 3, we can find that using the de-noised skeleton data, the recognition accuracy of all actions has been significantly improved, and an overall improvement of more than 5% was gained in the F1-score. At the same time, we can find that the proposed method achieved the highest accuracy in all five classes of actions. Among the other four manual feature extraction-based methods, the DNN classifier got the highest accuracy, while the Decision Tree classifier got the lowest. It is worth noting that for shaking hands, [39] has a smaller gap with other methods. A crucial reason is that [39] mainly focuses on the upper body features, especially the hands, so the actions of hands are more sensitive. Therefore, we can conclude that the methods based on manual feature extraction rely more on the professionalism of feature selection, and the omission of essential features will make the model invalid. On the contrary, the proposed method based on the LSTM network can automatically learn features from the time series ASD children’s skeleton data, then recognize the actions with higher accuracy.

## 4. Conclusions

In this paper, an action recognition method based on skeleton data and LSTM network was proposed for ASD children. It can recognize single or multiple ASD children’s action from the real-time or pre-recorded video. First, the OpenPose algorithm is used to extract skeleton data from the continuous image sequences, then proposed denoising methods eliminate the noise of the skeleton data. Next, the multi-person tracking model based on the skeleton data tracks multiple ASD children in the previous and subsequent frames. Finally, the LSTM network analyzes each ASD children’s skeleton data to recognize different actions.

Experimental results show that the recognition performance of the proposed method is better than traditional manual feature extraction methods, and the denoising of skeleton data can also improve the action recognition accuracy. It must be noted that our approach does not require special hardware, and the image taken by the conventional RGB camera can be used as the input of the system, which is almost non-invasive for ASD children. In addition, the end-to-end deep learning-based framework eliminates the need to manually extract features, allowing for the addition of new actions by just retraining the model with new data. This method can assist rehabilitation experts in recognizing the actions of ASD children automatically, to shorten the diagnosis cycle. Moreover, the number of stereotyped actions recognized by the proposed method can be used as an indicator to evaluate the effect of ASD rehabilitation.

In future research, more actions of ASD children and a larger dataset can be included. Also, we plan to transplant the proposed method to the embedded platform and conduct some real-time tests to verify the reliability of the system.

## Figures and Tables

**Table 1:** Dataset description.

Action No.	Action Name	No. of Frames	No. of Sequences
1	Sit	7245	189
2	Stand	7903	207
3	Squat	5997	178
4	Shake the body	10,613	251
5	Shake hands	9118	237
	Total	40,876	1062

**Table 2:** Summary of the computed features. Bold represents the features selected for experiments.

	Feature	Meaning
Method 1 [39]	$mean-\delta_{x}$, $mean-\delta_{y}$, $mean-\delta_{P}$	Mean velocity change in X co-ordinate, Y co-ordinate, and overall position of each key point
	$\sigma -\delta_{x}$, $\sigma -\delta_{y}$, $\sigma -\delta_{P}$	Standard deviation of velocity change in X co-ordinate, Y co-ordinate, and overall position of each key point
	$\max-\rho_{m}$	**Maximum magnitude of the autocorrelogram maxima computed for each of the three velocity measures tracked for each key point**
	$\mathrm{mean}-\rho_{m}$	**Mean deviation of the autocorrelogram maxima computed for each of the three velocity measures tracked for each key point**
	$\sigma -\rho_{m}$	**Standard deviation of the autocorrelogram maxima computed for each of the three velocity measures tracked for each key point**
	X	**Normalized joint positions**
Method 2	$X_{s}$	Concatenation of key points’ positions of N frames
	*H*	In $X_{s}$: Average height of the skeleton. It equals the length from Neck to Thigh.
	$V_{body}$	**In** $X_{s}$ **: Velocity of the body**
	X	**Normalized joint positions**
	$V_{joins}$	**In** X **: Velocity of all joints**

**Table 3:** P, R, and F1-score of five different methods under the original and de-noised skeleton data.

		Sit		Stand		Squat		Shake the Body		Shake Hands	
		Original	De-Noised	Original	De-Noised	Original	De-Noised	Original	De-Noised	Original	De-Noised
Method 1 + Decision Tree [39]	P	0.4498	0.524	0.8634	0.8899	0.5582	0.6169	0.7896	0.8417	0.7584	0.8235
	R	0.619	0.6762	0.7375	0.8083	0.6389	0.6889	0.6485	0.7091	0.7286	0.8
	F1-score	0.521	0.5905	0.7955	0.8471	0.5958	0.6509	0.7121	0.7697	0.7432	0.8116
Method 2 + linear SVM	P	0.5692	0.6109	0.8423	0.9095	0.6462	0.6667	0.8345	0.8955	0.8063	0.8339
	R	0.6857	0.7476	0.7792	0.8375	0.7	0.7556	0.7182	0.7788	0.7286	0.8071
	F1-score	0.622	0.6724	0.8095	0.872	0.672	0.7084	0.7719	0.8331	0.7655	0.8203
Method 2 + Random Forests	P	0.5774	0.6574	0.895	0.9334	0.6364	0.7005	0.8841	0.9263	0.8044	0.8856
	R	0.7286	0.7857	0.8167	0.8792	0.7389	0.8056	0.7394	0.8	0.7786	0.8517
	F1-score	0.6442	0.7158	0.8541	0.9055	0.6838	0.7494	0.8053	0.8585	0.7913	0.8711
Method 2 + DNN	P	0.5824	0.6798	0.9148	0.9437	0.6603	0.7438	0.9236	0.9613	0.8692	0.9145
	R	0.7571	0.819	0.85	0.9083	0.7667	0.8389	0.7697	0.8273	0.8071	0.8786
	F1-score	0.6584	0.7429	0.8812	0.9257	0.7095	0.7885	0.8397	0.8893	0.837	0.8962
Proposed method	P	0.6816	0.8448	0.9184	0.9835	0.7387	0.8852	0.9391	0.9835	0.9227	0.9821
	R	0.7952	0.9333	0.9375	0.9958	0.8167	0.9	0.7939	0.903	0.8964	0.9786
	F1-score	0.734	0.8868	0.9279	0.9896	0.7757	0.8925	0.8604	0.9415	0.9094	0.9803

## Data Availability

The data presented in this study are available on request from the corresponding author. The data are not publicly available due to the privacy of our participants and the requirements of our UCHRP (HR 400-2020).
